# Supplementary material for: Computational speed-up with a single qudit
Source: Sci Rep. 2015 Oct 8;5:14671. doi: 10.1038/srep14671 (PMC4597186; doi:10.1038/srep14671)
Supplement: Supplementary Information [file srep14671-s1.pdf]

Supplementary Information: Computational speed-up with a single qudit

Z. Gedik[1,*], I. A. Silva[2], B. Çakmak[1], G. Karpat[3,4], E. L. G. Vidoto[2], D. O. Soares-Pinto[2], E. R. deAzevedo[2], and F. F. Fanchini[3]

[1]Faculty of Engineering and Natural Sciences, Sabanci University, Tuzla, Istanbul, 34956, Turkey
[2]Instituto de Física de São Carlos, Universidade de São Paulo, Caixa Postal 369, 13560-970 São Carlos, São Paulo, Brazil
[3]Faculdade de Ciências, UNESP - Universidade Estadual Paulista, Bauru, São Paulo, 17033-360, Brazil
[4]Turku Center for Quantum Physics, Department of Physics and Astronomy, University of Turku, FIN-20014 Turku, Finland
[*]gedik@sabanciuniv.edu

ABSTRACT

1 Exponential of the phase operator

.

In case of a qutrit, we note that the transformations U_{f_2}, U_{f_3}, U_{f_5}, and U_{f_6} can be expressed in terms of the exponential of the phase operator given by

$$E = \sum_{m=-S}^{m=S} \mid S, m+1\rangle\langle S, m \mid .$$

where $\mid S,m\rangle$ is the simultaneous eigenstate of $\mathbf{S}^2$ and S_z operators, with eigenvalues $S(S+1)$ and m, respectively.[1] We can write the permutation transformations as $U_{f_2} = E$ and $U_{f_3} = E^\dagger$ with $S = 1$. Similarly, we have $U_{f_5} = E\Theta$ and $U_{f_6} = E^\dagger\Theta$, where Θ is the complex conjugation operator. Now, $\mid \psi_1\rangle$ is eigenstate of E and $E^\dagger$ with eigenvalues $\exp(-i2\pi/3)$ and $\exp(i2\pi/3)$, respectively. Therefore, the operations U_{f_1}, U_{f_2}, and U_{f_3}, leave $\mid \psi_1\rangle$ invariant, up to a phase. A similar argument holds for U_{f_4}, U_{f_5}, and U_{f_6} when they act on $\mid \psi_4\rangle$.

2 Spin Operators

A qutrit can be interpreted as half-way between one and two qubits. This interpretation can be made more concrete as follows. For a spin-1 system, squares of components of spin operator commute. For example, let us consider S_x^2 and S_y^2 where $[S_x^2, S_y^2] = 0$. Simultaneous eigenstates $\mid pq\rangle$ (with $p,q \in \{0,1\}$) of S_x^2 and S_y^2 resemble the computational basis of two qubits. However, due to the constraint $S_x^2 + S_y^2 + S_z^2 = 2$, allowed states are $\mid 01\rangle$, $\mid 10\rangle$, and $\mid 11\rangle$ with $\mid 00\rangle$ missing.

Spin operators provide an alternative interpretation of the algorithm. After the application of U_{f_k}, the qutrit is known to be either in state $\mid \psi_1\rangle$ or in state $\mid \psi_4\rangle$. Let us consider the Hermitian operator $M = \mid \psi_4\rangle\langle\psi_4 \mid - \mid \psi_1\rangle\langle\psi_1 \mid$. Possible states $\mid \psi_1\rangle$ and $\mid \psi_4\rangle$ are eigenstates of M with eigenvalues -1 and 1, respectively. We can write M, in terms of spin operators, as

$$M = \frac{i}{\sqrt{3}} \begin{pmatrix} 0 & -1 & 1 \\ 1 & 0 & -1 \\ -1 & 1 & 0 \end{pmatrix} = \sqrt{\frac{2}{3}} S_y - \frac{1}{\sqrt{3}}(S_x S_y + S_y S_x).$$

Therefore, if the qutrit enters a medium just after the U_{f_k} gate, so that the Hamiltonian operator is M, an energy measurement will give the information about the parity of f_k directly.

3 Results of the tomography for all operators

In Figs. 1 and 2, we display the tomography results after the final step of the algorithm, together with the fidelities to the theoretical prediction, for the implementation of positive cyclic permutation operations ((a) U_1, (b) U_2, (c) U_3, (d) U_4) and

negative cyclic permutation operations ((a) U_5, (b) U_6, (c) U_7, (d) U_8), respectively. We also present the entire description of each density matrix with the experimental error on their elements.

References

1. Vourdas A. SU(2) and SU(1,1) phase states Phys. Rev. A **41**, 1653 (1990).

(a)

F = 0.95

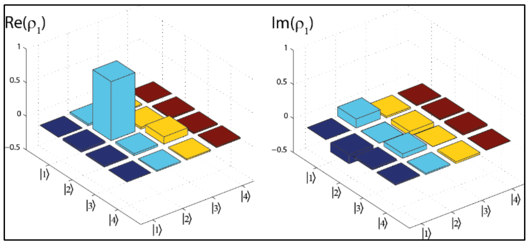

$$\begin{pmatrix} 0.00 & 0.03-0.02i & -0.01-0.01i & 0.00-0.03i \\ 0.03+0.02i & 0.91 & 0.024+0.012i & 0.03-0.03i \\ 0.01+0.01i & 0.024-0.01i & 0.070 & -0.02+0.00i \\ 0.00+0.03i & 0.03+0.03i & -0.02+0.00i & 0.02 \end{pmatrix} \pm \begin{pmatrix} 0.02 & 0.01 & 0.01 & 0.02 \\ 0.01 & 0.02 & 0.008 & 0.01 \\ 0.01 & 0.008 & 0.01 & 0.02 \\ 0.02 & 0.01 & 0.02 & 0.02 \end{pmatrix}$$

(b)

F = 0.97

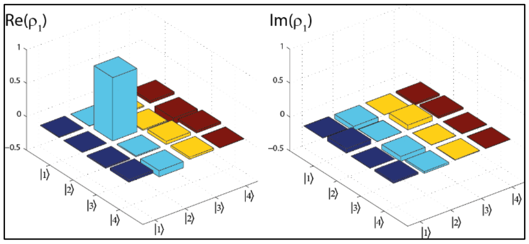

$$\begin{pmatrix} 0.01 & 0.00-0.06i & -0.02+0.00i & 0.03-0.02i \\ 0.00+0.06i & 0.95 & -0.016+0.070i & -0.09+0.03i \\ -0.02+0.00i & -0.016-0.070i & 0.05 & 0.03-0.01i \\ 0.03+0.02i & -0.09-0.03i & 0.03+0.01i & 0.00 \end{pmatrix} \pm \begin{pmatrix} 0.03 & 0.01 & 0.02 & 0.01 \\ 0.01 & 0.02 & 0.005 & 0.02 \\ 0.02 & 0.005 & 0.02 & 0.02 \\ 0.01 & 0.02 & 0.02 & 0.04 \end{pmatrix}$$

(c)

F = 0.98

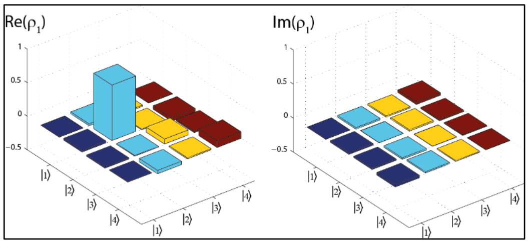

$$\begin{pmatrix} 0.00 & 0.03-0.02i & -0.02-0.02i & 0.00+0.04i \\ 0.03+0.02i & 0.83 & -0.011+0.021i & -0.05-0.02i \\ -0.02+0.02i & -0.011-0.021i & 0.07 & 0.02-0.01i \\ 0.00-0.04i & -0.05+0.02i & 0.02+0.01i & 0.10 \end{pmatrix} \pm \begin{pmatrix} 0.04 & 0.01 & 0.01 & 0.01 \\ 0.01 & 0.02 & 0.005 & 0.02 \\ 0.01 & 0.005 & 0.02 & 0.01 \\ 0.01 & 0.02 & 0.01 & 0.03 \end{pmatrix}$$

(d)

F = 0.95

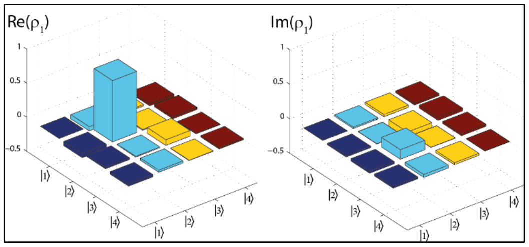

$$\begin{pmatrix} 0.00 & 0.03-0.02i & -0.02-0.02i & 0.00+0.04i \\ 0.03+0.02i & 0.83 & -0.011+0.021i & -0.05-0.02i \\ -0.02+0.02i & -0.011-0.021i & 0.07 & 0.02-0.01i \\ 0.00-0.04i & -0.05+0.02i & 0.02+0.01i & 0.10 \end{pmatrix} \pm \begin{pmatrix} 0.04 & 0.01 & 0.01 & 0.01 \\ 0.01 & 0.02 & 0.005 & 0.02 \\ 0.01 & 0.005 & 0.02 & 0.01 \\ 0.01 & 0.02 & 0.01 & 0.03 \end{pmatrix}$$

Figure 1. Tomography results and experimental errors after the final step of the algorithm, together with the fidelities to the theoretical prediction, for the implementation of the positive cyclic permutation operations: (a) U_1, (b) U_2, (c) U_3, (d) U_4.

(a)

F = 0.97

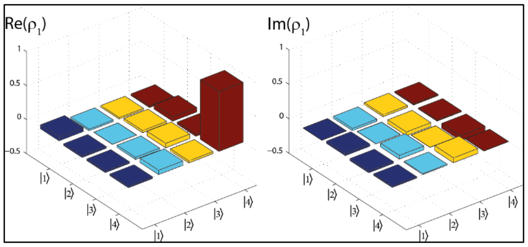

$$\begin{pmatrix} 0.06 & -0.02-0.03i & -0.02+0.03i & -0.02+0.02i \\ -0.02+0.03i & 0.00 & 0.031-0.054i & 0.06+0.01i \\ -0.02-0.03i & 0.031+0.054i & 0.07 & 0.03-0.07i \\ -0.02-0.02i & 0.06+0.01i & 0.03+0.07i & 0.90 \end{pmatrix} \pm \begin{pmatrix} 0.02 & 0.01 & 0.01 & 0.01 \\ 0.01 & 0.01 & 0.006 & 0.01 \\ 0.01 & 0.006 & 0.01 & 0.02 \\ 0.01 & 0.01 & 0.02 & 0.03 \end{pmatrix}$$

(b)

F = 0.96

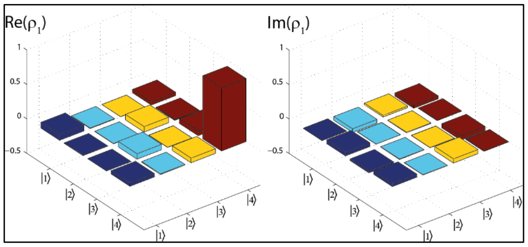

$$\begin{pmatrix} 0.08 & 0.01-0.05i & 0.00+0.02i & 0.05-0.04i \\ 0.01+0.05i & 0.00 & 0.079-0.007i & 0.01+0.00i \\ 0.00-0.02i & 0.079+0.007i & 0.00 & 0.07-0.06i \\ 0.05+0.04i & 0.01+0.00i & 0.07+0.06i & 0.91 \end{pmatrix} \pm \begin{pmatrix} 0.01 & 0.01 & 0.02 & 0.01 \\ 0.01 & 0.02 & 0.006 & 0.03 \\ 0.02 & 0.006 & 0.01 & 0.01 \\ 0.01 & 0.03 & 0.01 & 0.03 \end{pmatrix}$$

(c)

F = 0.95

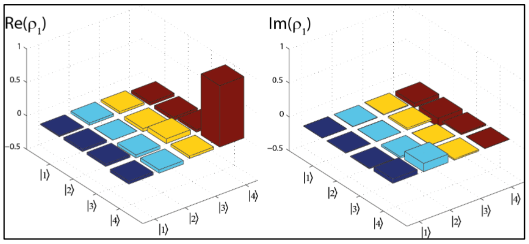

$$\begin{pmatrix} 0.01 & 0.04-0.01i & 0.03+0.01i & -0.04-0.06i \\ 0.04+0.01i & 0.00 & -0.042+0.02i & -0.05-0.12i \\ 0.03-0.01i & -0.042-0.02i & 0.07 & 0.04-0.02i \\ -0.04+0.06i & -0.05+0.12i & 0.04+0.02i & 0.92 \end{pmatrix} \pm \begin{pmatrix} 0.04 & 0.01 & 0.03 & 0.02 \\ 0.01 & 0.03 & 0.007 & 0.03 \\ 0.03 & 0.007 & 0.03 & 0.01 \\ 0.02 & 0.03 & 0.01 & 0.05 \end{pmatrix}$$

(d)

F = 0.97

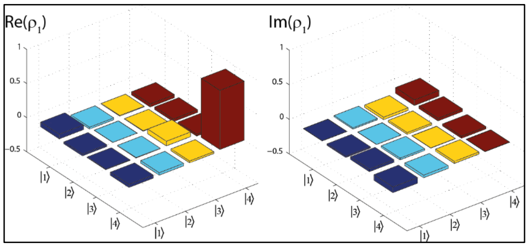

$$\begin{pmatrix} 0.07 & -0.03+0.02i & -0.01-0.04i & -0.04+0.08i \\ -0.03-0.02i & 0.00 & -0.005-0.027i & -0.04+0.04i \\ -0.01+0.04i & -0.005+0.027i & 0.07 & -0.03-0.03i \\ -0.04-0.08i & -0.04-0.04i & -0.03+0.03i & 0.86 \end{pmatrix} \pm \begin{pmatrix} 0.02 & 0.02 & 0.01 & 0.01 \\ 0.02 & 0.02 & 0.006 & 0.02 \\ 0.01 & 0.006 & 0.02 & 0.02 \\ 0.01 & 0.02 & 0.02 & 0.03 \end{pmatrix}$$

Figure 2. Tomography results and experimental errors after the final step of the algorithm, together with the fidelities, for the implementation of the negative cyclic permutation operations: (a) U_5, (b) U_6, (c) U_7, (d) U_8.
